# Adhesive-free adhesion between heat-assisted plasma-treated fluoropolymers (PTFE, PFA) and plasma-jet-treated polydimethylsiloxane (PDMS) and its application

**DOI:** 10.1038/s41598-018-36469-y

**Published:** 2018-12-24

**Authors:** Yuji Ohkubo, Katsuyoshi Endo, Kazuya Yamamura

**Affiliations:** Graduate School of Engineering, Osaka University, 2-1 Yamadaoka, Suita Osaka, 565–0871 Japan

## Abstract

Conventional low-temperature plasma treatment was reported to minimally improve the adhesion property of polytetrafluoroethylene (PTFE), whereas heat-assisted plasma (HAP) treatment significantly improved the same. An unvulcanized rubber was previously used as an adherent for PTFE. This study aimed to achieve strong adhesive-free adhesion between PTFE and vulcanized polydimethylsiloxane (PDMS) rubber. As-received vulcanized PDMS rubber did not adhere to HAP-treated PTFE, and as-received PTFE did not adhere to vulcanized rubber of plasma-jet (PJ) treated PDMS rubber; however, HAP-treated PTFE strongly adhered to vulcanized PJ-treated PDMS rubber, and both PTFE and PDMS exhibited cohesion failure in the T-peel test. The surface chemical compositions of the PTFE and PDMS sides were determined using X-ray photoelectron spectroscopy. The strong PTFE/PDMS adhesion was explained via hydrogen and covalent bond formation (C–O–Si and/or C(=O)–O–Si) between hydroxyl (C–OH) or carboxyl (C(=O)–OH) groups of the HAP-treated PTFE. This process was also applied to adhesive-free adhesion between a tetrafluoroethylene–perfluoroalkylvinylether copolymer (PFA) and PDMS; subsequently, a translucent PFA/PDMS assembly with strong adhesion was realized together with the PTFE/PDMS assembly. Strong adhesive-free adhesion between fluoropolymers (PTFE, PFA) and vulcanized PDMS rubber without using any adhesives and graft polymer was successfully realized upon plasma treatment of both the fluoropolymer and PDMS sides. Additionally, a PDMS sheet, which was PJ-treated on both sides, was applied to strongly adhere fluoropolymers (PTFE, PFA) to materials such as metal and glass. PJ-treated PDMS was used as an intermediate layer rather than a strong adhesive, achieving PTFE/PDMS/metal and PTFE/PDMS/glass assemblies. The PTFE/PDMS, PDMS/metal, and PDMS/glass adhesion strengths exceeded 2 N/mm.

## Introduction

Polytetrafluoroethylene (PTFE) comprises only CF_2_ chains and a typical fluoropolymer. While this fluoropolymer offers several advantages, such as good water and oil repellency, high chemical resistance, weather resistance, and good sliding properties, it does not readily adhere to other types of materials because of its low surface energy and weak boundary layer^1,2^. To overcome its extremely poor adhesion properties, chemical etching using corrosive solutions containing sodium has long been utilized^3–5^. However, chemical etching has several disadvantages, including malodour, toxicity to humans, a high environmental load, and PTFE surface coloration. Therefore, an alternative method that does not require corrosive solutions has long been needed. Specifically, dry processes such as ion irradiation and plasma treatment are potentially suitable alternatives to chemical etching because these present almost no danger to humans together with a low environmental load. Ion irradiation is performed under low pressure such as 1.3 × 10^−2^ Pa. However, plasma treatment can be performed under a wide range of pressures from low to atmospheric pressure, e.g., 1.0 × 10^5^ Pa; thus, plasma treatment has possibility of having no vacuum evacuation system. This pressure difference indicates that plasma treatment is potentially more suitable than ion irradiation for practical purposes, especially large area continuous processing. However, conventional plasma treatment results in minimal improvement in the adhesion properties of PTFE. Although some reports described on indirect adhesion between PTFE and other types of materials using adhesives^6,7^ and/or graft polymers^8–11^, hardly any reports mentioned on adhesive-free adhesion without graft polymerization. We considered the parameters for the plasma treatment conditions such as pressure, plasma treatment time, and polymer surface temperature. Finally, we developed heat-assisted plasma (HAP) treatment and realized strong adhesive-free adhesion between PTFE and unvulcanized rubber without graft polymerization^12^. On analyzing the PTFE surface, it was concluded that HAP was central to both introducing oxygen-containing functional groups (O–C=O, C=O, C–O) on the PTFE surface and increasing the surface hardness through C–C crosslinking formation and etching during plasma treatment to improve the adhesion properties of PTFE^13^. Additionally, several types of unvulcanized rubber containing different rubber compounding agents, such as crosslinking and reinforcing agents, were prepared; the unvulcanized rubber was then adhered to HAP-treated PTFE to determine which rubber compounding agent was the most effective in improving the adhesion strength. Consequently, it was found that hydrophilic SiO_2_ powder, having a silanol group (Si–OH) that functions as a reinforcing agent, contributed to the high adhesion strength between HAP-treated PTFE and unvulcanized rubber^14^. In previous studies^12–14^, unvulcanized rubbers were employed as adherents. In the present study, we focus on vulcanized rubber as an adherent to PTFE. We selected vulcanized polydimethylsiloxane (PDMS) as a vulcanized rubber. It is reported that when a PDMS surface is modified by plasma, corona discharge, and ultraviolet (UV) treatments, then silanol groups (Si–OH) are readily generated^15–17^. Additionally, PDMS is widely used in the medical industry, especially as a material for microfluid chips^18–21^. Adhesive bonding between PDMS surfaces and adhesive-free adhesion between PDMS surfaces have already been established, and related techniques are reported in many research articles^22–24^. Moreover, both adhesive bonding and adhesive-free adhesion between PDMS and other types of materials (resins such as PMMA, PP, and PE^25–27^; metals such as Cu, Fe, Pt, Au, and Al^27,28^; and glass^28–30^) were also reported. However, these articles rarely reported on adhesion between PDMS and fluoropolymers, or the adhesion strength between PDMS and PTFE was too low for practical use^27,31,32^. In medical and food industries, adhesives are unlikely to be employ because they are regarded as contaminations. Thus, adhesive-free adhesion is essential in the medical and food industries. Adhesive-free adhesion between PDMS and PTFE using tetrapodal ZnO fillers were reported and the adhesion strength increased upon addition of the fillers, but the maximum adhesion strength was below 0.3 N/mm^33^. Thus, the effect of shape of fillers was not enough to strongly adhere PDMS to fluoropolymers. In this study, we aim to achieve strong adhesive-free adhesion based on chemical interaction between vulcanized PDMS and fluoropolymers containing PTFE.

## Results

### Surface chemical composition analysis of plasma-treated PDMS and plasma-treated PTFE using X-ray photoelectron spectroscopy (XPS)

**Figure 1:**
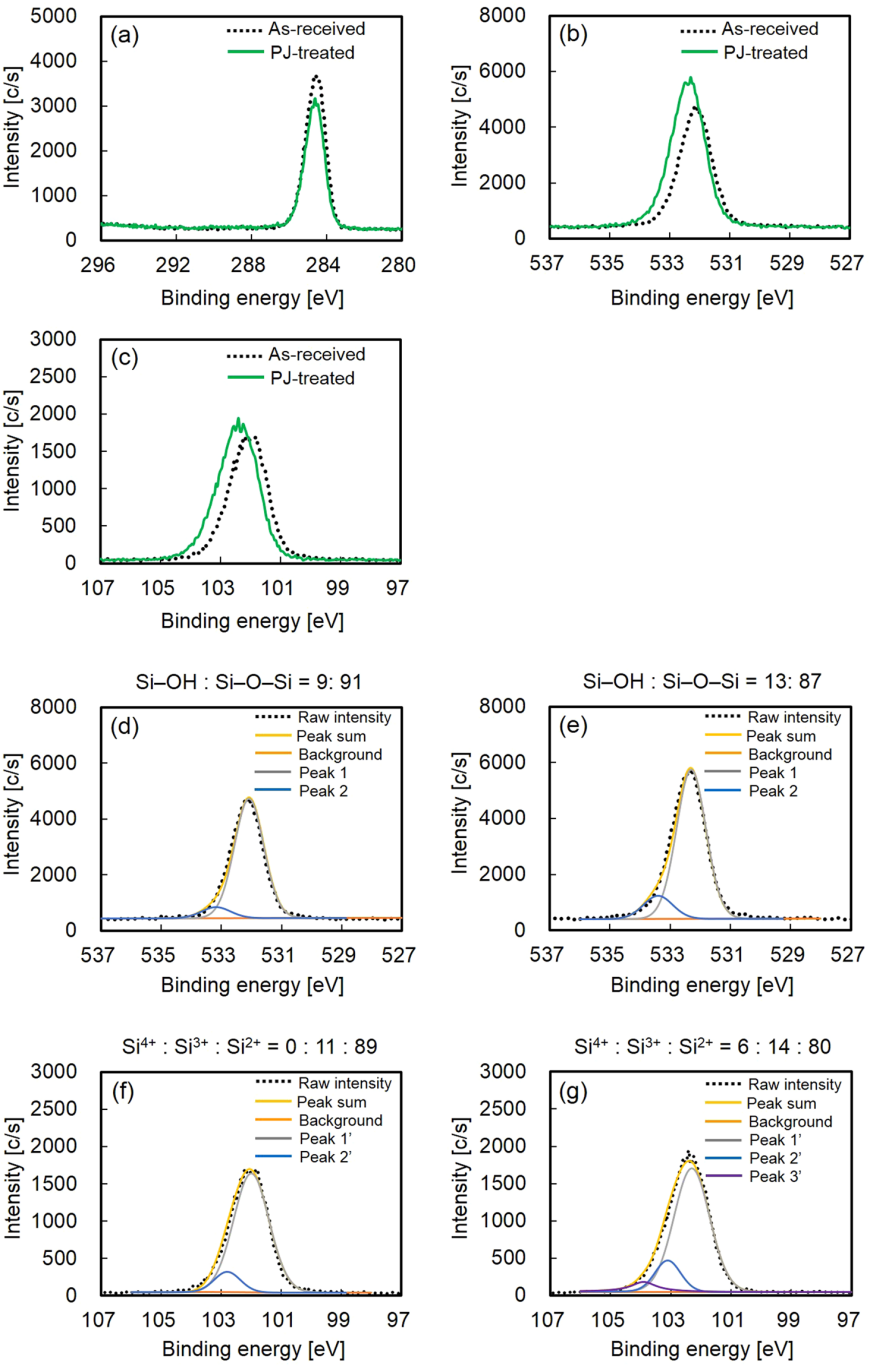
XPS spectra of the PDMS samples: (**a**) C1s-XPS spectra of the PDMS samples before and after PJ treatment, (**b**) O1s-XPS spectra of the PDMS samples before and after PJ treatment, (**c**) Si2p-XPS spectra of the PDMS samples before and after PJ treatment, (**d**) peak resolution of O1s-XPS spectrum of the PDMS sample before PJ treatment, (**e**) peak resolution of O1s-XPS spectrum of the PDMS sample after PJ treatment, (**f**) peak resolution of Si2p-XPS spectrum of the PDMS sample before PJ treatment, and (**g**) peak resolution of Si2p-XPS spectrum of the PDMS sample after PJ treatment. Peaks 1 and 2 indicate Si–O–Si and Si–OH, respectively. Peaks 1′, 2′ and 3′ indicate Si^2+^: –[Si(CH_3_)_2_–O–]–, Si^3+^: –[Si(CH_3_)(OH)–O–]–, and Si^4+^: –[Si(OH)_2_–O–]–, respectively.

Figure 1a–1c shows the XPS spectra of the PDMS samples before and after plasma-jet (PJ) treatment. The intensity of the peak indexed to CH_3_ decreased after PJ treatment, resulting in C–Si bond scission and desorption of CH_3_ on the PJ-treated PDMS surface (Fig. 1a). No peaks indexed to C–O (286.5 eV)^34,35^, C=O (288.0 eV)^34,35^, or O–C(=O) (289.2 eV)^34,35^ were observed for the PJ-treated PDMS surface (Fig. 1a). The intensity of the O1s peak increased and shifted to higher binding energy after PJ treatment, indicating an oxidation reaction on the PJ-treated PDMS surface (Fig. 1b). The intensity of the Si2p peak also increased and shifted to higher binding energy after PJ treatment, also indicating an oxidation reaction on the PJ-treated PDMS surface (Fig. 1c). Furthermore, peak resolution of O1s-XPS and Si2p-XPS spectra of as-received and PJ-treated PDMS samples was conducted. Figure 1d,1e shows peak resolution of the O1s-XPS spectra of the PDMS samples before and after PJ treatment. Peaks 1 and 2 indicate Si–O–Si and Si–OH, respectively^36,37^. The as-received PDMS sample had the ratio Si–OH: Si–O–Si = 9:91. This also indicated that the surface of the as-received PDMS sample used in this study originally contained ca. 10% Si–OH. The PJ-treated PDMS sample had the ratio Si–OH: Si–O–Si = 13:87. These results indicated that Si–OH increased after PJ treatment. Figure 1f,1g shows peak resolution of the Si2p-XPS spectra of the PDMS samples before and after PJ treatment. Peaks 1′, 2′ and 3′ indicate Si^2+^: –[Si(CH_3_)_2_–O–]–, Si^3+^: –[Si(CH_3_)(OH)–O–]–, and Si^4+^: –[Si(OH)_2_–O–]–, respectively^36,37^. As-received PDMS had the ratio –[Si(OH)_2_–O–]–: –[Si(CH_3_)(OH)–O–]–: –[Si(CH_3_)_2_–O–]– = 0:11:89. This Si2p-XPS result together with the O1s-XPS result also indicated that the surface of the as-received PDMS sample used in this study originally contained ca. 10% Si–OH. PJ-treated PDMS had the ratio –[Si(OH)_2_–O–]–: –[Si(CH_3_)(OH)–O–]–: –[Si(CH_3_)_2_–O–]– = 6:14:80. These results also suggested that Si–OH increased after PJ treatment. Previous articles^15,36,37^ reported Si–C scission and CH_3_ desorption upon plasma treatment, followed by formation of Si–O and/or Si–OH upon reaction between Si radicals and oxygen atoms or OH^−^ ions in plasma. These reports are consistent with our study.

**Figure 2:**
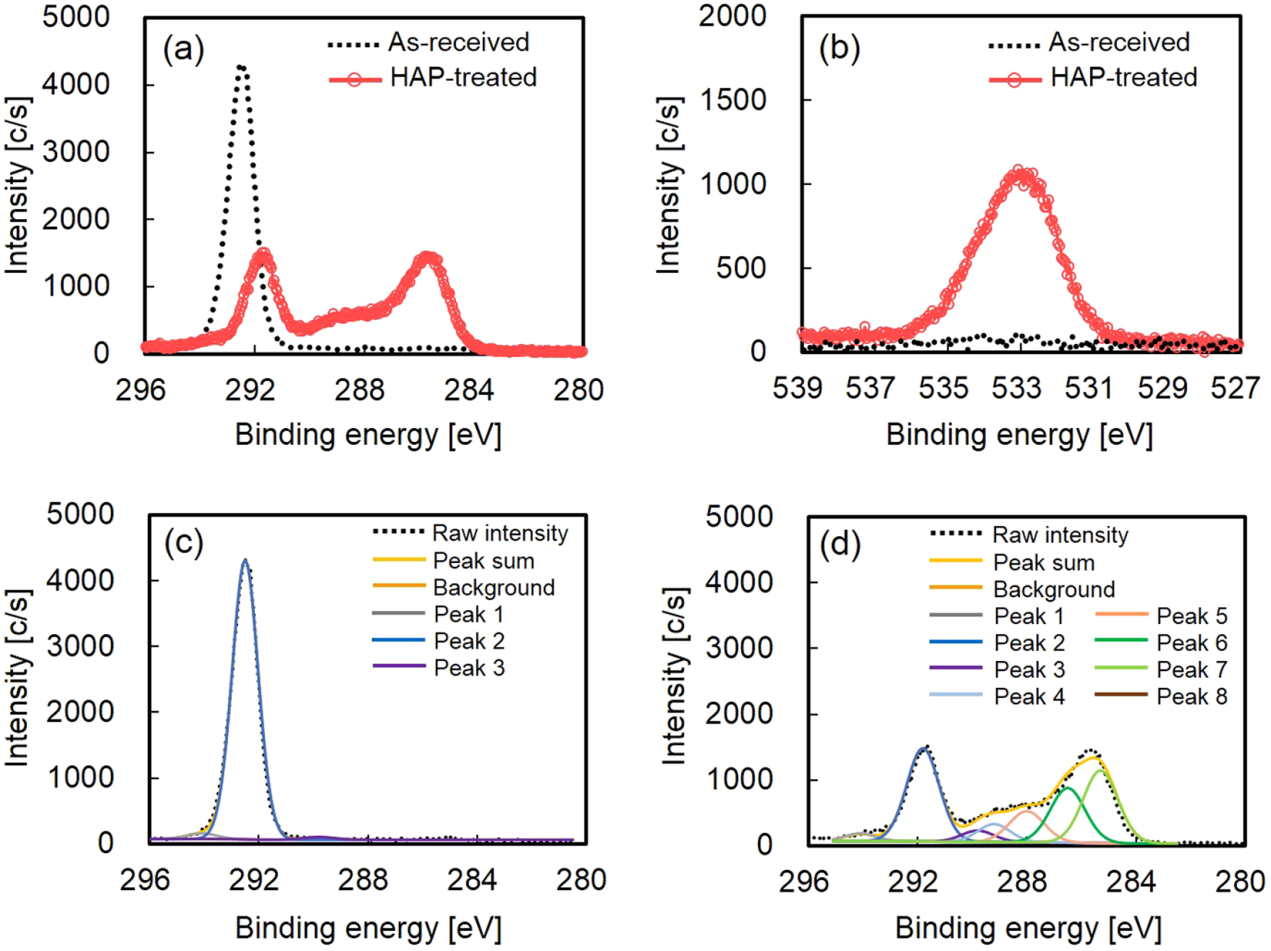
XPS spectra of the PTFE samples: (**a**) C1s-XPS spectra of the PTFE samples before and after HAP treatment, (**b**) O1s-XPS spectra of the PTFE samples before and after HAP treatment, (**c**) peak resolution of C1s-XPS spectrum of the PTFE sample before HAP treatment, and (**d**) peak resolution of C1s-XPS spectrum of the PTFE sample after HAP treatment. Peaks 1–8 indicate CF_3_, CF_2_, C–F, O–C=O, C=O, C–O, C–C, and C=C, respectively.

Figure 2a,2b shows the XPS spectra of the PTFE samples before and after HAP treatment. The as-received PTFE sample exhibited only a peak indexed to CF_2_ at ca. 292 eV, as shown in Fig. 2a. However, the HAP-treated PTFE sample had peaks indexed to not only fluorine-containing functional groups (CF_3_, CF_2_, C–F)^13,34,35,38^ at ca. 292 eV but also oxygen-containing functional groups (O–C=O, C=O, C–O)^34,35^ at ca. 289–286 eV and carbon groups (C–C, C=C)^39^ at ca. 286–284 eV, as shown in Fig. 2a. Additionally, the as-received PTFE sample exhibited no peaks, whereas the HAP-treated PTFE sample exhibited a broad peak at ca. 531–537 eV, as shown in Fig. 2b. These results indicated that the PTFE surface was oxidized upon HAP treatment. Furthermore, peak resolution was conducted for C1s-XPS spectra of as-received and HAP-treated PTFE samples. Figure 2c,2d shows peak resolution of the C1s-XPS spectra of the PTFE samples before and after HAP treatment. Peaks 1–8 indicate CF_3_, CF_2_, C–F, O–C=O, C=O, C–O, C–C, and C=C, respectively. Table 1 shows the functional group ratios on the PTFE samples before and after HAP treatment, calculated from the C1s-XPS spectra shown in Fig. 2c,2d. These results indicated that both C–C crosslinking and the generation of oxygen-containing functional groups occurred on the HAP-treated PTFE surface.

**Table 1:** Ratios of functional groups on the PTFE samples before and after HAP treatment, calculated from C1s-XPS spectra shown in Fig. 2c,2d.

Peak No.	Functional group		As-received	Unit [%]
				HAP treatment
Peak 1	CF_3_	294.1 eV	2.3	2.2
Peak 2	CF_2_	292.5* or 291.8** eV	96.6 *	31.3**
Peak 3	C–F	289.8 eV	1.1	3.7
Peak 4	O–C=O	289.2 eV	0.0	5.9
Peak 5	C=O	288.0 eV	0.0	11.0
Peak 6	C–O	286.5 eV	0.0	19.7
Peak 7	C–C	285.3 eV	0.0	26.2
Peak 8	C=C	284.3 eV	0.0	0.0

* and ** indicate that the obtained XPS spectra of as-received and HAP-treated PTFE samples were referenced to peaks indexed to –CF_2_– at 292.5 eV^34,35^ and 291.8 eV^13,38^, respectively.

### Adhesion strength between plasma-treated fluoropolymers and PDMS

**Figure 3:**
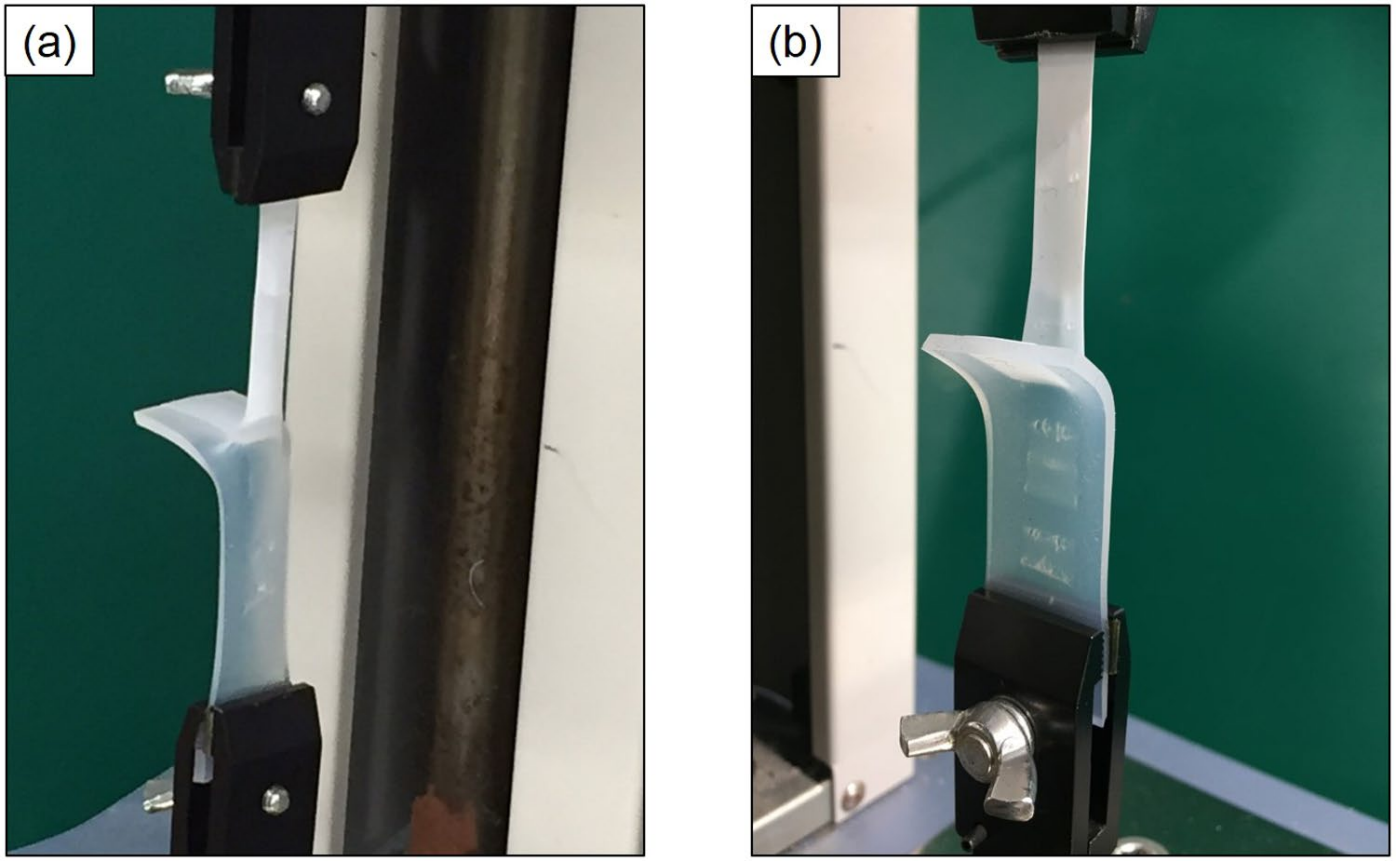
Photographs of PTFE/PDMS assembly during the T-peel test: (**a**) front side and (**b**) back side.

**Figure 4:**
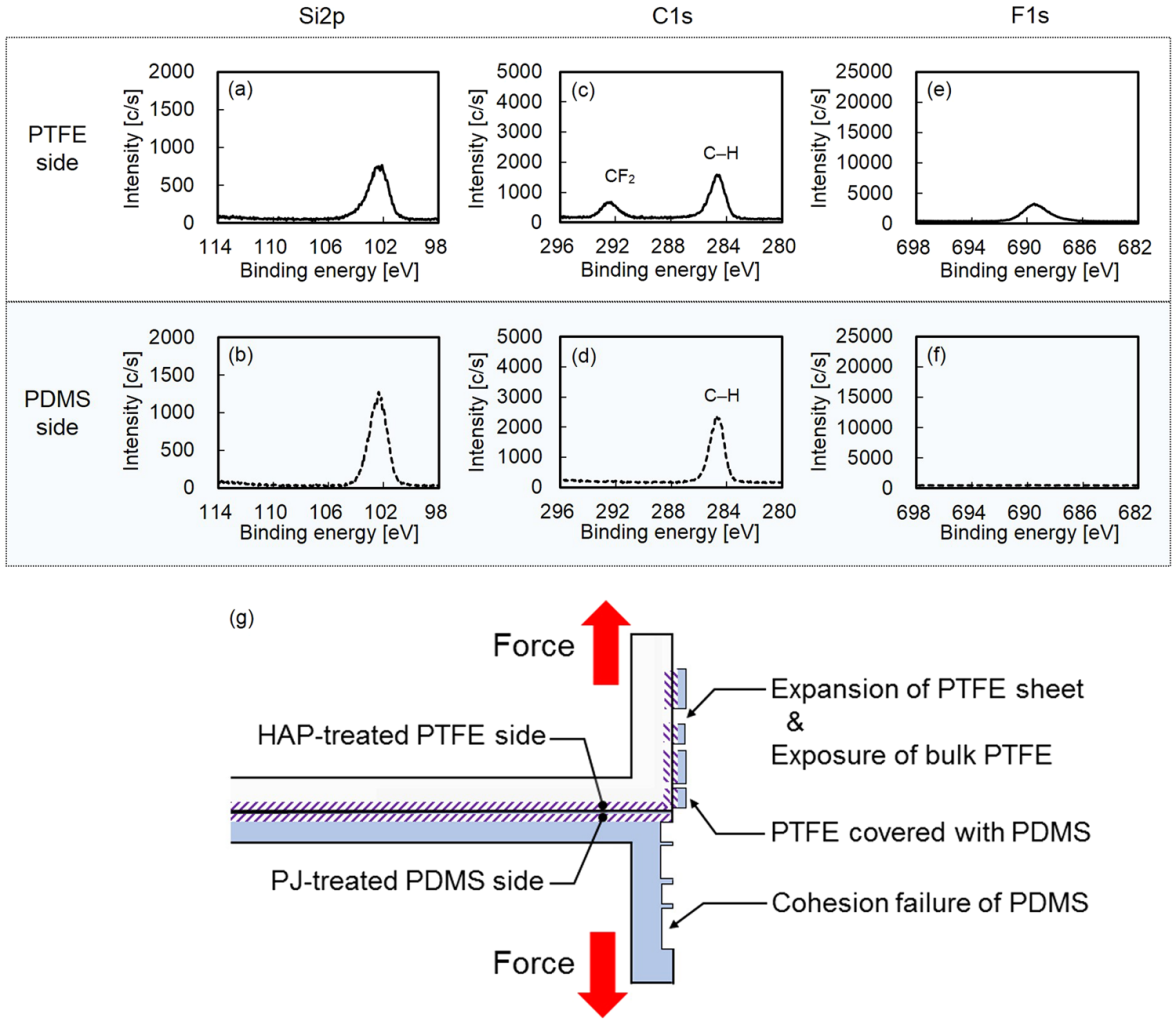
XPS spectra of the peeled surfaces of the PTFE/PDMS assembly sample in which both sides of PDMS and PTFE sheets were plasma-treated: (**a**) Si2p-XPS spectrum of the PTFE side, (**b**) Si2p-XPS spectrum of the PDMS side, (**c**) C1s-XPS spectrum of the PTFE side, (**d**) C1s-XPS spectrum of the PDMS side, (**e**) F1s-XPS spectrum of the PTFE side, (**f**) F1s-XPS spectrum of the PDMS side, and (**g**) schematic of peeled surfaces.

Table 2 shows the sample preparation conditions and adhesion strengths of the PTFE/PDMS assembly. When the as-received PDMS or PTFE samples were used, the adhesion strength of the PTFE/PDMS assembly was 0 N/mm. Thus, the vulcanized PDMS sample required PJ treatment to strongly adhere to plasma-treated PTFE, although unvulcanized natural rubber (NR) containing hydrophilic SiO_2_ powder did not require plasma treatment. When both sides of PDMS and PTFE were plasma-treated, the adhesion strength of the PTFE/PDMS assembly was 2.6 N/mm, which indicated drastic increase of adhesion strength. The load-displacement curve of the PTFE/PDMS assembly was shown in Supplementary Information Fig. S1(a). Figure 3 shows the photographs of the PTFE/PDMS assembly during the T-peel test, where both sides of the PDMS and PTFE sheets were plasma-treated. Both the PDMS and PTFE sheets were extended during the T-peel test (Fig. 3a). Figure 3b shows that PDMS which was strongly adhered to the PTFE surface was transferred to the PTFE side after the peel test. Figure 4 shows the XPS spectra and schematic of the peeled surfaces of the PTFE/PDMS assembly sample. Si was detected on both the PTFE and PDMS sides (Fig. 4a,4b). C–H but not CF_2_ was detected on the PDMS side, while the peak indexed to C–H had a higher intensity than that indexed to CF_2_ on the PTFE side (Fig. 4c,4d). Thus, most of the PTFE surface was covered with PDMS after the peel test and a small amount of CF_2_ was detected at 292.5 eV but not at 291.8 eV, because the PTFE sheet was extended during the T-peel test because of strong adhesion of the interface between PTFE and PDMS, after which bulk PTFE appeared on the surface, as illustrated in Fig. 4g. These results indicated cohesion failure of both PDMS and PTFE occurred when HAP-treated PTFE adhered strongly to PJ-treated PDMS. In summary, the adhesion strength of PTFE/PDMS interface was higher than breaking strengths of both PDMS and PTFE. For comparison, when PTFE was plasma-treated at low temperature (below 100 °C), the PTFE was thermally compressed to PJ-treated PDMS, and the PTFE/PDMS adhesion strength was 0.1 N/mm, which was extremely low. The effect of heating during plasma treatment was also confirmed during this study. HAP treatment was essential for obtaining high adhesion strength in the PTFE/PDMS assembly.

**Table 2:** Sample preparation conditions and the adhesion strengths of PTFE/PDMS assembly.

Sample condition		Adhesion strength [N/mm]
PDMS	PTFE	
×	×	0.0 ± 0.0
×	○**	0.0 ± 0.0
○*	×	0.0 ± 0.0
○*	○**	2.6 ± 0.2***

“×” denotes no plasma treatment, and “○” denotes plasma treatment.

*PJ treatment.

**HAP treatment.

***Cohesion failure of PDMS rubber and/or PTFE sheet during T-peel test.

**Figure 5:**
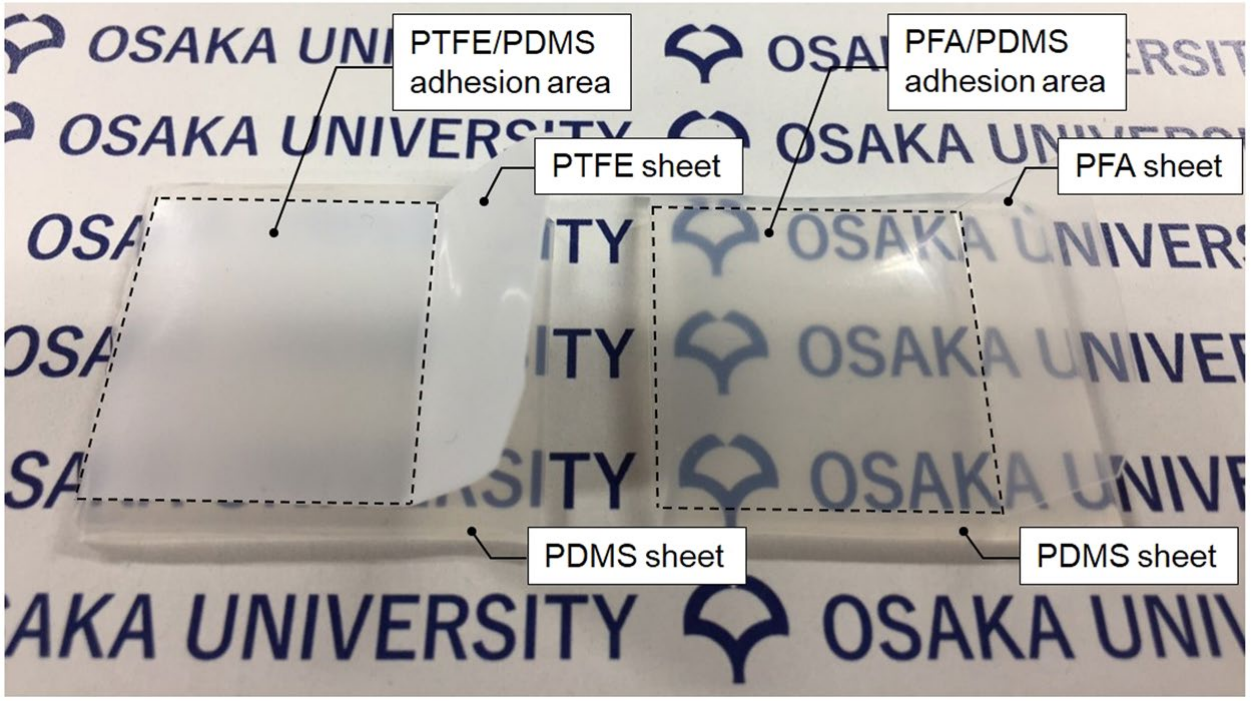
Photographs of PTFE/PDMS and PFA/PDMS assemblies on a paper showing the school badge of Osaka University. The thicknesses of the PTFE, PFA, and PDMS sheets were 0.2, 0.1 and 2 mm, respectively.

Rather than PTFE, tetrafluoroethylene–perfluoroalkylvinylether copolymer (PFA) sheet was HAP-treated at 19.1 W/cm^2^ before preparing a PFA/PDMS assembly by thermal compression of the PJ-treated PDMS and HAP-treated PFA sheets. The adhesion strength of the PFA/PDMS assembly, in which the thicknesses of the PFA and PDMS sheets were 0.1 and 2 mm, respectively, exceeded 2 N/mm, which indicated strong adhesive-free adhesion between the PDMS and PFA sheets. Figure 5 shows the photographs of PTFE/PDMS and PFA/PDMS assemblies on a paper showing the school badge of Osaka University. Although the badge was not observed under the PTFE/PDMS assembly, it was clearly observed under the PFA/PDMS assembly. The optical transparency of the PFA/PDMS assembly would allow observation of liquid flow in a PFA/PDMS assembly hose and/or tube, which would have high flexibility, chemical resistance, and weather resistance.

### Strong adhesive-free adhesion between PTFE and other types of materials (metal and glass) via PJ-treated PDMS

**Figure 6:**
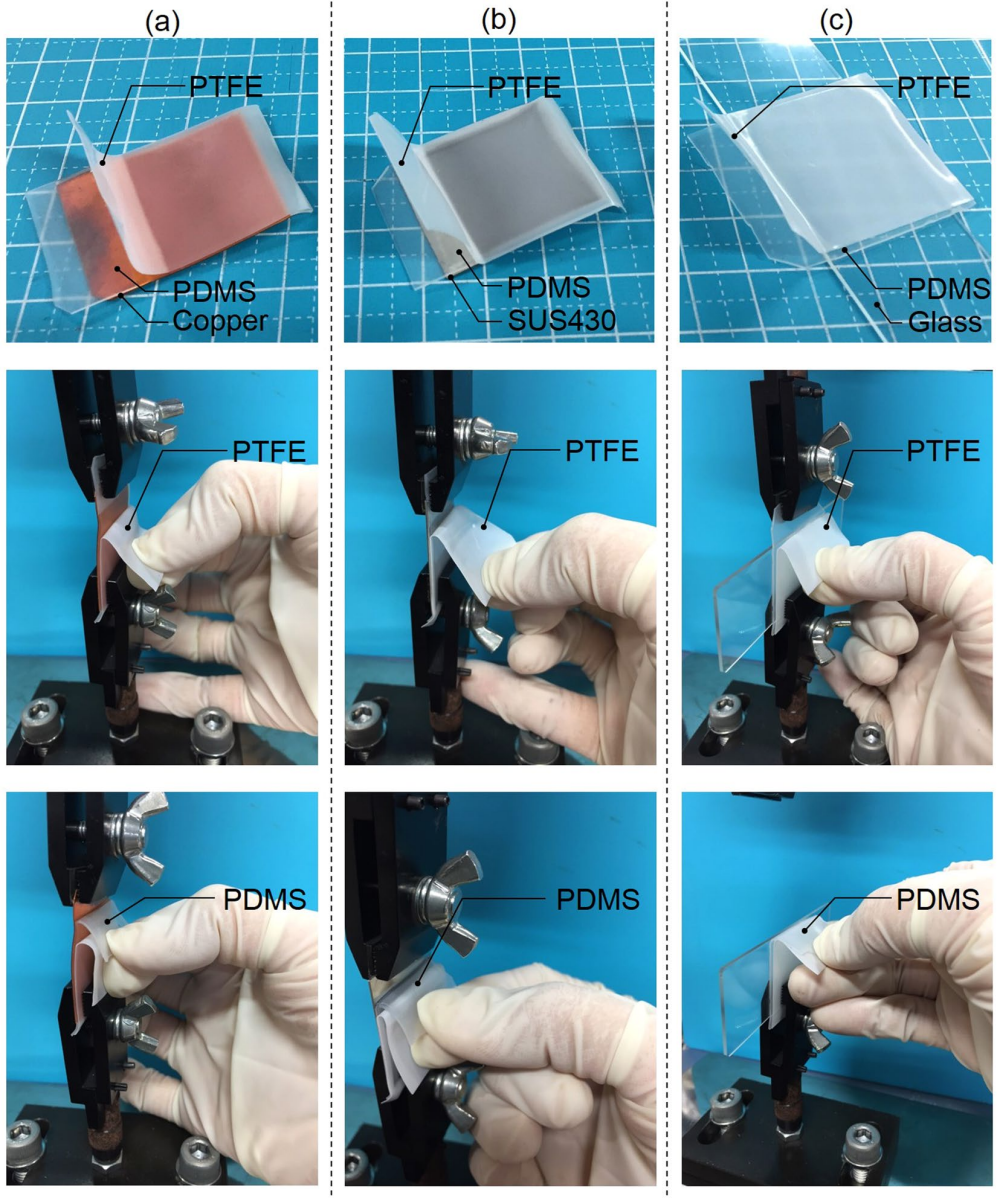
Photograph of three-layer assemblies of (**a**) PTFE/PDMS/Cu, (**b**) PTFE/PDMS/SUS430, and (**c**) PTFE/PDMS/glass. When the PTFE or PDMS sheet was jerked and shaken, no peeling occurred at the interfaces of PTFE/PDMS, PDMS/Cu, PDMS/SUS430, and PDMS/glass. It was shown that PJ-treated PDMS could be used as an alternative to strong adhesives to stick fluoropolymers to other types of materials. The adhesion strengths of the interfaces of PTFE/PDMS, PDMS/Cu, PDMS/SUS430, and PDMS/glass were measured using a 90° peel test, and all the adhesion strengths exceeded 2 N/mm.

As described in the Introduction section, it was previously reported that plasma-treated PDMS could strongly adhere to several materials such as metal and glass^27–30^. We attempted adhesive-free adhesion between fluoropolymers (PTFE, PFA) and other types of materials such as metal and glass by combining of a technique reported previously with that developed in this study. We confirmed whether PJ-treated PDMS readily adhered to metal (copper and stainless steel) or glass. The load-displacement curve of the assemblies of Cu/PDMS, SUS430/PDMS, and PDMS/glass were shown in Supplementary Information Fig. S1(b)–(d). The photographs of Cu/PDMS, SUS430/PDMS, and PDMS/glass were shown in Supplementary Information Fig. S2(a)–(c). We then prepared three-layer assemblies: PTFE/PDMS/metal and PTFE/PDMS/glass, as shown in Fig. 6. It was shown that strong adhesive-free adhesion between fluoropolymers (PTFE, PFA) and other types of materials such as metal and glass was readily realized by PJ treatment of both sides of a PDMS sheet.

## Discussion

In this study, vulcanized PDMS rubber was employed as an adherent for fluoropolymers, rather than unvulcanized rubber, which contains hydrophilic SiO_2_ powder containing Si–OH groups. Although unvulcanized rubber adhered strongly to HAP-treated fluoropolymers without PJ treatment of the rubber in a previous study^14^, vulcanized rubber did not adhere to HAP-treated fluoropolymers without PJ treatment in the present study. When a vulcanized PDMS rubber was subjected to PJ treatment, we realized strong adhesive-free adhesion between the HAP-treated fluoropolymer and vulcanized PDMS. It was clear that this was achieved by plasma treatment of both sides of the fluoropolymer and vulcanized rubber.

**Figure 7:**
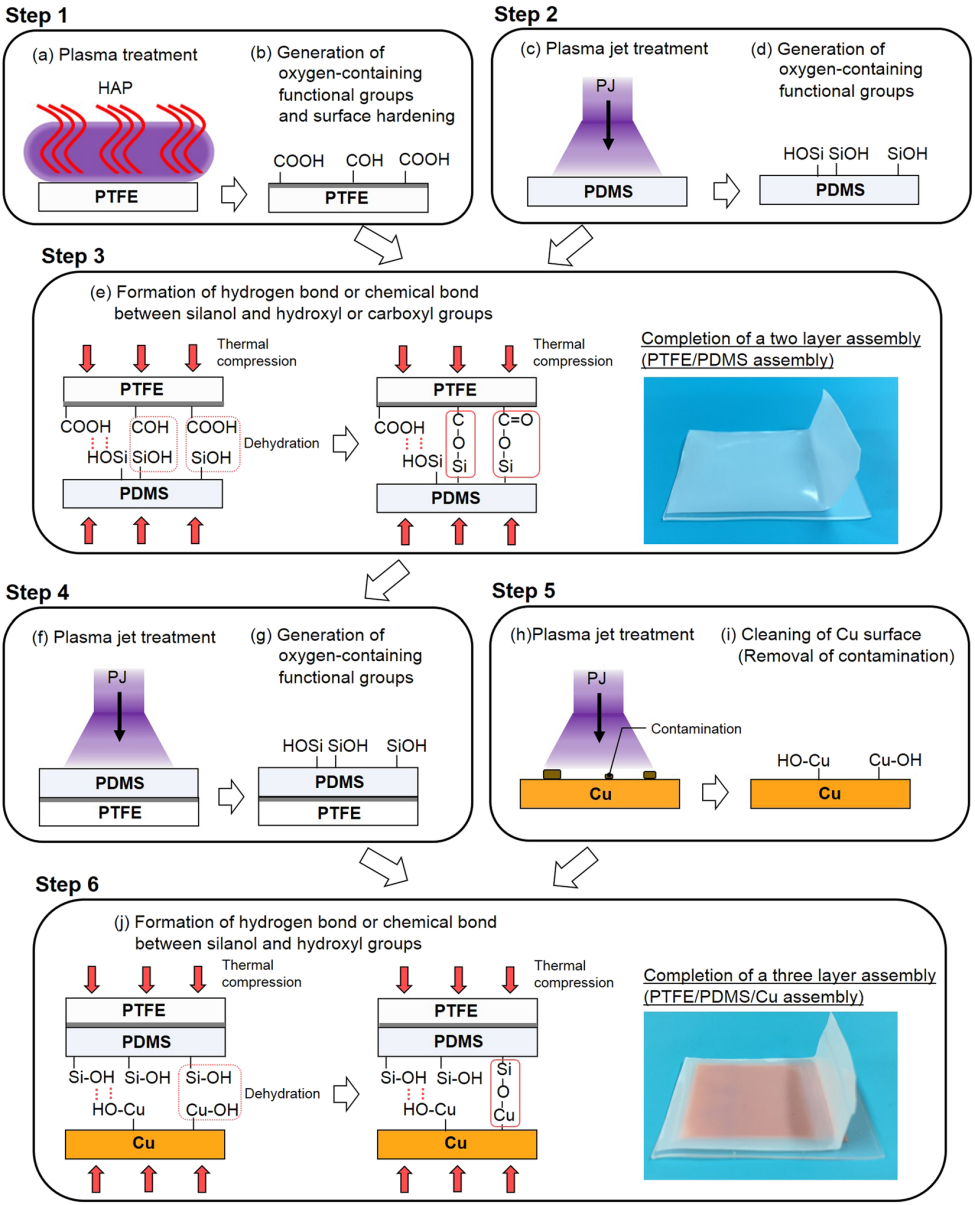
Proposed model for strong adhesion and preparation procedure for a two-layer assembly such as PTFE/PDMS (steps 1–3) and a three-layer assembly such as PTFE/PDMS/Cu (steps 1–6). PFA/PDMS, PFA/PDMS/glass, PTFE/PDMS/glass, and PTFE/PDMS/SUS430 assemblies were prepared in the same way.

Figure 7 shows a proposed model for strong adhesive-free adhesion as well as a preparation procedure for a two-layer assembly such as PTFE/PDMS and a three-layer assembly such as PTFE/PDMS/Cu. Firstly, a PTFE sheet is HAP-treated above 200 °C; oxygen-containing functional groups (C(=O)–OH, C–OH) are then generated and surface hardening occurs on the PTFE surface via HAP treatment. Secondly, a PDMS sheet is PJ-treated, and silanol groups (Si–OH) are generated on the PDMS surface. Thirdly, thermal compression of the HAP-treated PTFE sheet and the PJ-treated PDMS sheet is conducted, forming hydrogen bonds between silanol groups on the PJ-treated PDMS surface and hydroxyl or carboxyl groups on the HAP-treated PTFE surface. Additionally, dehydration condensation occurs between silanol and hydroxyl or carboxyl groups, forming C–O–Si and/or C(=O)–O–Si bonds. Considering that cohesion failure of both PDMS and PTFE occurred when PJ-treated PDMS and HAP-treated PTFE were thermally compressed, it is reasonable to assume that not only hydrogen bonds but also covalent bonds (C–O–Si and/or C(=O)–O–Si) were formed. Fourthly, the side of the PDMS surface of the PTFE/PDMS assembly is PJ-treated, generating silanol groups (Si–OH) on the PDMS surface. Fifthly, a copper plate is also PJ-treated to clean its surface; contamination is then removed, and hydroxyl groups (Cu–OH) are generated on the Cu surface. Finally, thermal compression of the PJ-treated PDMS/PTFE assembly and the PJ-treated Cu plate is performed, resulting in formation of hydrogen bonds between silanol groups on the PJ-treated PDMS/PTFE surface and hydroxyl groups on the PJ-treated Cu surface. Additionally, dehydration condensation occurs between silanol and hydroxyl groups, resulting in Si–O–Cu bond formation. Considering that cohesion failure of PDMS occurred when PJ-treated PDMS and PJ-treated Cu were thermally compressed, it is reasonable to assume that not only hydrogen bonds but also covalent bonds (Si–O–Cu) are formed.

This technique for achieving strong adhesive-free adhesion between PDMS and fluoropolymer adds several functions, such as high chemical resistance, water and oil repellency, antifouling and sliding properties, to PDMS, and elasticity to fluoropolymers. Additionally, three-layer assemblies such as PTFE/PDMS/metal and PTFE/PDMS/glass were successfully prepared by PJ treatment of both sides of a PDMS sheet. This demonstrated that PJ-treated PDMS can adhere fluoropolymers to several materials such as metal and glass as desired. In summary, this technique for achieving adhesive-free adhesion between PDMS combined with fluoropolymer will expand the application range of PDMS and fluoropolymers will be useful in various fields, especially the medical and food industries, in which adhesives are unsuitable.

## Methods

### Materials

An addition-crosslinked PDMS sheet (KE541U + C25A/B, HATADA, hardness 40°) was cut into 35 mm × 50 mm pieces, which was used as a silicone rubber specimen. A PDMS sheet with a thickness of 2 mm was mainly used, but a PDMS sheet with a thickness of 0.5 mm was also used for realization at high optical transparency. Commercially available PTFE sheet (NITOFLON^®^No. 900UL, Nitto Denko; thickness: 0.2 mm) was cut into 45 mm × 70 mm pieces, which was used as a fluoropolymer specimen. The color of PTFE sheet is white and PTFE has no optical transparency due to its high crystallinity. Conversely, the PFA sheet originally has optical transparency as well as PDMS sheet. Therefore, the PFA sheet (AF-0100, DAIKIN INDUSTRIES; thickness: 0.1 mm) was used only when high transparency was required. Copper foil (Cu, 99.9%, CU-113263, Nilaco Corporation; thickness: 0.050 mm) was cut into 30 mm × 25 mm pieces, which was used as a metal foil specimen. A pure copper plate (Cu, 99.96%, CU-113381, Nilaco Corporation; thickness: 0.20 mm) was also cut into 30 mm × 25 mm pieces, which was used as a Cu plate specimen. Stainless steel foil (SUS304, TS200-200-005, IWATA MFG; thickness: 0.05 mm) was cut into 30 mm × 25 mm pieces, which was used as a SUS foil specimen. A stainless steel plate (SUS430, HSO531, Hikari; thickness: 0.5 mm) was also cut into 30 mm × 25 mm pieces, which was used as a SUS plate specimen. A glass slide (S7213, Matsunami Glass Ind.) with 76 mm × 26 mm × 1 mm was used without cutting.

### Sample preparation by plasma treatment

Prior to PJ treatment, the PDMS sheets were washed sequentially with acetone (99.5%, Kishida Chemical) and pure water for 1 min each in an ultrasonic bath (US-4R, AS- ONE). The washed PDMS sheets were then dried using an air gun containing N_2_ gas (99.99%, Iwatani Fine Gas). The washed and dried PDMS sheets were then plasma-treated using open-air-type PJ treatment equipment (Tough Plasma FPE-20, FUJI CORPORATION), but not HAP-treated. The gap between the irradiation hole and the surface of the PDMS sheet was 10 mm; a mixture of N_2_ gas (99.99%, Iwatani Fine Gas) and air gas (N_2_/O_2_ = 79%/21%, Iwatani Fine Gas) was used as a process gas for plasma generation, and the flow rates of N_2_ and air gases were 29.7 and 0.3 L/min, respectively. The PDMS sheet was placed on a movable stage and fixed using double-sided polyimide tape (10-mm width, No. 4390, 3 M Japan), then the stage was moved at 8 mm/s during PJ treatment. The number of scan operations was only one in this study. To confirm whether the PJ treatment conditions were suitable, two PJ-treated PDMS sheets were prepared under the same PJ treatment conditions; the adhesion strength of the PDMS/PDMS assembly was then measured using a T-peel test. Cohesion failure of the PDMS occurred during the T-peel test, as shown in Supplementary Information Fig. S2(d). It was confirmed that these PJ conditions were suitable for PDMS.

It was previously reported that low-temperature plasma treatment barely improved the adhesion properties of PTFE, whereas HAP treatment significant improved them^12,13^. On the basis of these reports, the surface of the PTFE sheet was modified via HAP treatment in the present study. The detailed surface treatment procedure for a PTFE sheet using HAP has been previously reported^13^; hence, it is described only briefly here. Prior to HAP treatment, PTFE sheets as well as PDMS sheets were washed with acetone and pure water using an ultrasonic bath (US-4R, AS- ONE) then dried using an air gun containing N_2_ gas (99.99%, Iwatani Fine Gas). The washed and dried PTFE sheets were then HAP-treated at 19.1 W/cm^2^ for 600 s using He gas (99.99%, Iwatani Fine Gas) at atmospheric pressure in a custom-made chamber system (Meisyo Kiko)^12–14^. During HAP treatment, the surface temperature of the PTFE samples was measured with a digital radiation thermometer system (FT-H40K and FT-50A, Keyence); it was confirmed that the maximum surface temperature exceeded 200 °C during HAP treatment at 19.1 W/cm^2^ for 600 s. To confirm whether the HAP treatment conditions were suitable, an HAP-treated PTFE sheet and unvulcanized NR containing hydrophilic SiO_2_ powder were prepared; the adhesion strength of the PTFE/NR assembly was then measured using a T-peel test. Cohesion failure of the NR occurred during the T-peel test, as shown in Supplementary Information Fig. S2(e). It was thus confirmed that these HAP conditions were suitable for PTFE^14^.

A glass slide was used without washing or PJ treatment. Cu foils, Cu plate, SUS foils, and SUS plates as well as PDMS sheets were washed in an ultrasonic bath (US-4R, AS- ONE) then dried using an air gun containing N_2_ gas (99.99%, Iwatani Fine Gas). The washed and dried Cu foils, Cu plate, SUS foils, and SUS plates were also PJ-treated to clean their surfaces. The gap between the irradiation hole and the surface of the Cu foils, Cu plate, SUS foils, or SUS plates was 10 mm. The stage was moved at 0.8 mm/s during PJ treatment for Cu foils, Cu plate, SUS foils, or SUS plates. The number of scan operations was five for Cu foils, Cu plate, SUS foils, and SUS plates.

### Surface chemical composition analysis

XPS measurements were performed using a scanning XPS spectrometer (Quantum-2000, Ulvac-Phi) with a monochromated Al-*K*α source. All the XPS spectra were obtained below 5 × 10^−6^ Pa. The photoelectron take-off angle was 45°, and the X-ray irradiation area was Ø100 μm. Narrow scan XPS spectra of Si2p, C1s, O1s, and F1s were collected at 95–115 eV, 275–300 eV, 525–545 eV, and 680–700 eV, respectively, with a pass energy of 23.50 eV and a step size of 0.05 eV. The cumulative number of measurements was three. During an XPS measurement, the samples were irradiated with a low-speed electron beam and an Ar ion beam to achieve charge neutralization. The obtained XPS spectra of the PDMS samples were referenced to the peak indexed to C–Si–O–Si and/or C–H at 284.6 eV^36,37^, and the obtained XPS spectra of as-received and HAP-treated PTFE samples were referenced to peaks indexed to –CF_2_– at 292.5 eV^34,35^ and 291.8 eV^13,38^, respectively. The main peak of –CF_2_– in the C1s-XPS spectra shifted toward lower binding energy for plasma-treated PTFE due to surface charging^13,40^.

### Adhesion strength test for a two-layer assembly

Firstly, the HAP-treated PTFE sample was placed on the PJ-treated PDMS sheets in a mold such that the plasma-treated surfaces faced each other. Secondly, the PTFE/PDMS assembly was compressed without adhesive at 180 °C and 10 MPa for 10 min using a hot-pressing machine (AH-2003, AS-ONE). Thirdly, the PTFE/PDMS assembly was restored to room temperature. Fourthly, the adhesion strength of the PTFE/PDMS assembly was measured using a T-peel test by combining of a digital force gauge (ZP-200N, Imada) and an electric-driven stand (MX-500N, Imada). The T-peel test was conducted at room temperature of 25 ± 5 °C, and the humidity was not controlled. The sweep rate was 60 mm/min. Finally, the average adhesion strength was calculated by dividing the average tensile strength by the width of the PTFE (ca. 10 mm). To verify the reproducibility, three samples were prepared under the same conditions. A PFA/PDMS assembly was also prepared, and its adhesion strength was measured as for the PTFE/PDMS assembly.

### Adhesion strength test for a three-layer assembly

The first to third steps for a three-layer assembly were the same as those for a two-layer assembly. Fourthly, a PJ-treated PDMS/PTFE sample was placed on a PJ-treated Cu plate in a mold so that the plasma-treated surfaces faced each other. Fifthly, the PTFE/PDMS/Cu assembly was compressed without adhesive at 180 °C and 5 MPa for 10 min using a hot-pressing machine (AH-2003, AS-ONE). Sixthly, the PTFE/PDMS/Cu assembly was restored to room temperature. Seventhly, the PTFE and PDMS sheet on the Cu plate was cut to a width of 10 mm. Eighthly, the Cu plate was fixed on the electric-driven stand (MX-500N, Imada) using two stainless steel bars, then both the PTFE and PDMS sheets were simultaneously grasped and pulled up when measuring the adhesion strength of the PDMS/Cu interface with a 90° peel test. The 90° peel test was conducted under the same conditions of temperature and humidity as T-peel test. The sweep rate was also 60 mm/min. When the adhesion strength of the PTFE/PDMS interface was measured using a 90° peel test, the Cu plate was fixed on the electric-driven stand using two stainless steel bars, and then only the PTFE sheet was pulled up. The PTFE/PDMS/SUS430 assembly was prepared as for the PTFE/PDMS/Cu assembly. The PFA/PDMS/glass and PTFE/PDMS/glass assemblies were prepared as for the PTFE/PDMS/Cu assembly except for the fifth step, in which the pressure was decreased from 5 to almost 0 MPa (the empty weight of the PFA/PDMS or PTFE/PDMS assembly) because the glass slide was easily broken. The adhesion strengths of PFA/PDMS/glass, PTFE/PDMS/glass, and PTFE/PDMS/SUS430 were measured as for the PTFE/PDMS/Cu assembly. Peeling tests were conducted at least two times for each three-layer assembly to verify the reproducibility.

## Electronic supplementary material

Supplementary-Information
