# Supplementary material for: Adhesive-free adhesion between heat-assisted plasma-treated fluoropolymers (PTFE, PFA) and plasma-jet-treated polydimethylsiloxane (PDMS) and its application
Source: Sci Rep. 2018 Dec 24;8:18058. doi: 10.1038/s41598-018-36469-y (PMC6305381; doi:10.1038/s41598-018-36469-y)
Supplement: Supplementary file 1 — Supplementary-Information [file 41598_2018_36469_MOESM1_ESM.pdf]

Supplementary Information

Adhesive-free adhesion between heat-assisted plasma-treated fluoropolymers (PTFE, PFA) and plasma-jet-treated polydimethylsiloxane (PDMS) and its application

Yuji Ohkubo,[1,*] Katsuyoshi Endo,[1] and Kazuya Yamamura[1]

[1]Graduate School of Engineering, Osaka University, 2-1 Yamadaoka, Suita, Osaka 565-0871, Japan

Contents

● Load-displacement curves ········ Fig. S1
● Photographs ········ Fig. S2

Figure S1. Load-displacement curves of (a) PTFE/PDMS for T-peel test: 10.3-mm width, (b) Cu/PDMS for T-peel test: 14.5-mm width, (c) SUS430/PDMS for T-peel test: 12.7-mm width, and (d) PDMS/glass for 90 degree peel test: 10.0-mm width. For PTFE/PDMS sample, the average adhesion strength were calculated by dividing the load in the stable region by the width of the sample. For Cu/PDMS, SUS430/PDMS, and PDMS/glass samples, the load in the stable region was not observed because PDMS sheet or glass plate was broken. Therefore, the adhesion strength were calculated by dividing the maximum load by the width of the sample.

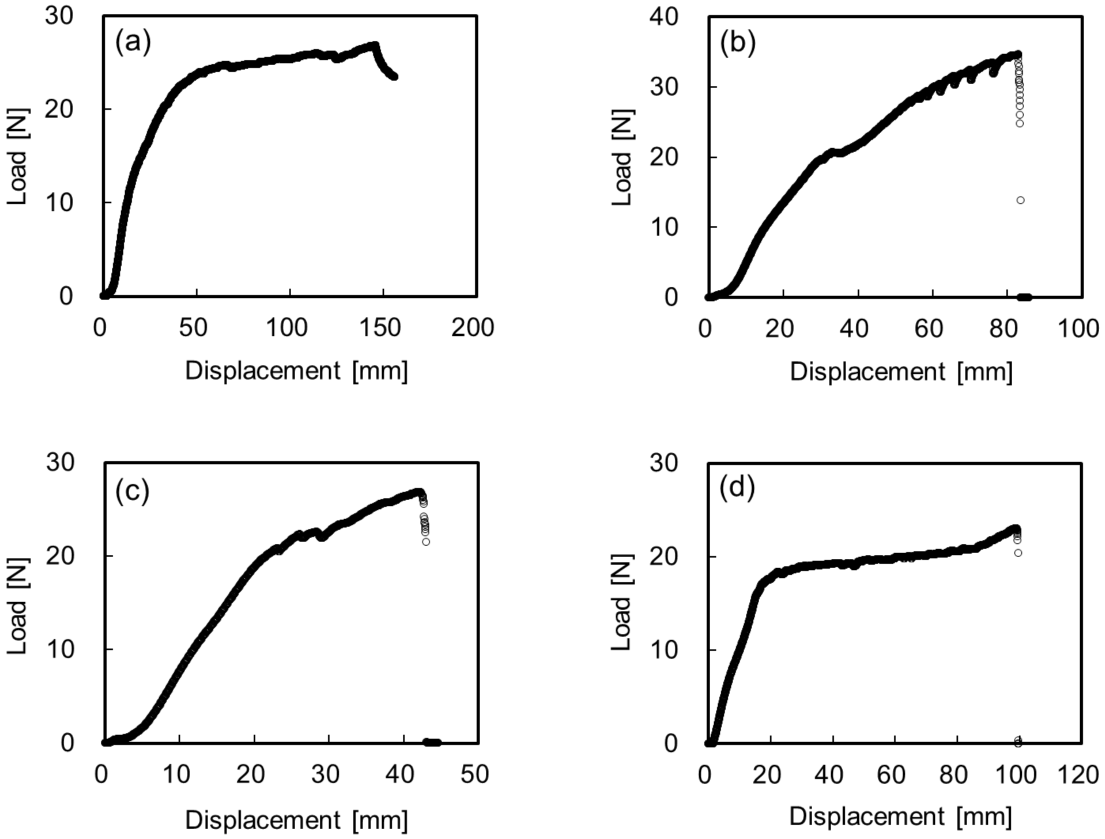

Figure S2. Photographs of (a) PDMS/copper (Cu) foil, (b) PDMS/stainless steel (SUS304) foil, (c) PDMS/glass, (d) PDMS/PDMS, and (e) HAP-treated PTFE/natural rubber (NR) after adhesion confirmation test. When the adhesion strengths of the interfaces of PDMS/Cu and PDMS/SUS304 were measured using a T-peel test, both the adhesion strengths exceeded 2 N/mm, and cohesion failures of PDMS occurred. When the adhesion strength of the PDMS/glass interface was measured using a 90° peel test, the adhesion strength also exceeded 2 N/mm, and the glass slide was broken. It was confirmed that PJ-treated PDMS adhered strongly to metal and glass without use of adhesives. Although no adhesives were used in this study, the PDMS/PDMS adhesion strength also exceeded 2 N/mm, and cohesion failure of PDMS occurred. It was confirmed that PJ-treated PDMS had strong adhesion ability prior to the adhesion strength test for fluoropolymers. The PTFE/NR adhesion strength also exceeded 2 N/mm, and cohesion failure of NR occurred. It was confirmed that HAP-treated PTFE had strong adhesion ability prior to the adhesion strength test for PDMS.

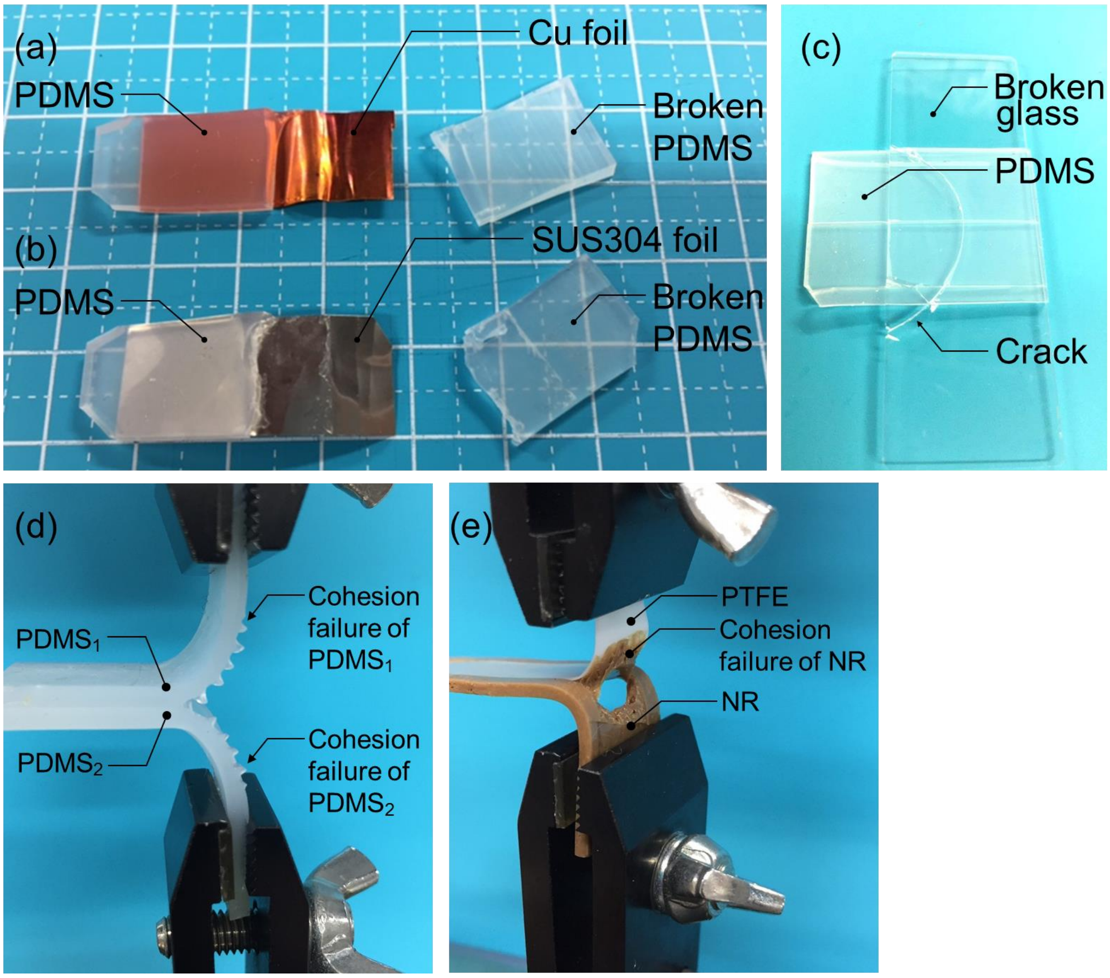
(a)
PDMS
Cu foil
Broken PDMS
(b)
PDMS
SUS304 foil
Broken PDMS
(c)
Broken glass
PDMS
Crack
(d)
PDMS$_1$
PDMS$_2$
Cohesion failure of PDMS$_1$
Cohesion failure of PDMS$_2$
(e)
PTFE
Cohesion failure of NR
NR
